# The Influence of 2.45 GHz Wi-Fi Exposure Duration on Sperm Quality and Testicular Histopathology: An Exploration of Peroxidative Injury

**DOI:** 10.3390/antiox14020179

**Published:** 2025-02-04

**Authors:** Norazurashima Jamaludin, Siti Fatimah Ibrahim, Farah Hanan Fathihah Jaffar, Aini Farzana Zulkefli, Khairul Osman

**Affiliations:** 1 Centre of Diagnostic, Therapeutic and Investigation Study, Faculty of Health Sciences, Universiti Kebangsaan Malaysia (UKM), Jalan Raja Muda Abdul Aziz, Kuala Lumpur 50300, Malaysia; norazurashima@ilkkm.edu.my; 2 Department of Anatomy & Physiology, Institut Latihan Kementerian Kesihatan Malaysia (ILKKM), Jalan Hospital, Sungai Buloh, Selangor 47000, Malaysia; 3 Department of Physiology, Faculty of Medicine, Universiti Kebangsaan Malaysia (UKM), Jalan Yaacob Latif, Bandar Tun Razak, Cheras, Kuala Lumpur 56000, Malaysia; timi@ukm.edu.my (S.F.I.); farahhanan@ukm.edu.my (F.H.F.J.); ainifarzana@ppukm.ukm.edu.my (A.F.Z.)

**Keywords:** duration, Wi-Fi, histology, malondialdehyde, sperm quality

## Abstract

Concerns have arisen about the impact of wireless technology on male fertility, particularly regarding the duration of 2.45 GHz Wi-Fi radiation exposure. This study examines the influence of various exposure durations on sperm parameters and testicular histopathology, focusing on malondialdehyde as an oxidative stress marker. Twenty-four Sprague Dawley rats were exposed for eight weeks, after which their sperm concentration, motility, and viability and testicular histopathology were assessed. Malondialdehyde levels were measured using an Enzyme-Linked Immunosorbent Assay. One-way ANOVAs with Tukey’s post hoc tests were conducted for the sperm concentration, motility, and viability; the seminiferous epithelium height; and malondialdehyde. The Kruskal–Wallis H test was used for the Johnsen Score and seminiferous tubule diameter. The results indicated that 4 h of exposure to 2.45 GHz radiation induced oxidative stress and adversely affected sperm parameters and the testicular ultrastructure. Gradual recovery was observed at 8 h, with further improvement at 24 h, suggesting the activation of cell repair mechanisms. This was supported by significant changes in testicular organ coefficients, indicating potential recovery. Our findings suggest that Wi-Fi exposure reduces sperm fertility potential, with the body showing limited capacity for complete recovery from the damage.

## 1. Introduction

In an era characterized by the omnipresence of wireless technology, Wi-Fi networks have seamlessly integrated into our daily lives, reshaping how we communicate and connect with the world [1]. The convenience and prevalence of Wi-Fi technology continue to surge, prompting an increasing urgency to comprehend its potential implications for human health. Among the myriad of concerns surrounding this technology, one issue of profound scientific interest and societal significance has emerged: the influence of Wi-Fi radiation, specifically operating at the commonly used 2.45 GHz frequency, on the male reproductive system [2,3].

Numerous potential mechanisms have been explored, with oxidative stress consistently being a key contributor. This stress stems from an imbalance between reactive oxygen species (ROS) and the biological system’s ability to neutralize reactive intermediates effectively [4]. One significant consequence of oxidative stress is lipid peroxidation, during which various byproducts are generated, including malondialdehyde (MDA) [4,5]. MDA is renowned for its mutagenic properties and is widely used to indicate oxidative radicals [6,7]. MDA is a naturally occurring result of the peroxidation of polyunsaturated fatty acids. Lipid peroxidation typically occurs on polyunsaturated fatty acids due to numerous linkages with reactive hydrogen. This aldehyde generates free radicals and inhibits hydrogen atoms from becoming liquid free radicals, which are then oxidized to create peroxyl radicals. Peroxyl radicals reacting with other polyunsaturated fatty acids lead to a continuous chain reaction, as described by Jadoon et al. in 2017 [5]. Testes and sperm possess a membrane composed of polyunsaturated fatty acids, rendering them very susceptible to damage from peroxidation. Akbari et al., Akdag et al., and Ajayi et al. reported that elevated levels of MDA indicate heightened lipid peroxidation. Hence, MDA can function as a comprehensive index of lipid peroxidation levels [4,8,9].

Recently, attention has shifted towards a pivotal aspect of this inquiry: the duration of exposure to radiofrequency electromagnetic radiation (RF-EMR) and its association with sperm parameters [3,10]. The widespread use of wireless technology and prolonged periods of RF-EMR exposure have sparked significant concerns regarding its impact on male reproductive health [2,10]. Several findings consistently underscore the crucial role of exposure duration in understanding the potential effects of Wi-Fi radiation on the male reproductive system [11,12,13]. Studies consistently demonstrate that prolonged exposure to 2.45 GHz Wi-Fi radiation has more pronounced impacts on male reproductive health. More prolonged exposure durations correlate with more significant changes in essential parameters related to male reproductive health. Therefore, it is imperative to consider the temporal aspect of these effects. Studies examining the correlation between exposure duration and sperm parameters have produced ambiguous results [3,6,10]. While several studies have documented adverse effects on sperm cell lines and in rats following short-term RF-EMR exposure, others have observed no significant impact [14,15]. Similarly, research on the consequences of prolonged exposure has yielded inconsistent findings, with some suggesting detrimental effects and others revealing no notable changes in sperm characteristics [2,8].

Sperm parameters, such as motility and viability, are significantly affected by Wi-Fi radiation exposure, potentially due to peroxidative damage, as evidenced by increased MDA levels [2,6,12]. However, it is crucial to acknowledge that the relationship between the Wi-Fi radiation exposure duration and male reproductive health is complex and not yet fully understood. Therefore, this study explores the Wi-Fi exposure durations commonly reported by Malaysians, based on data from the Malaysian Communications and Multimedia Commission (MCMC) 2020 [16]: less than 4 h for light users, 8 to 15 h for intermediate users, and more than 15 h for heavy users. It examines how these exposure times may contribute to peroxidative damage, indicated by MDA, and their effects on male reproductive health. By focusing on these typical usage patterns, the study offers valuable insights into the potential risks and consequences of increasing reliance on wireless technology.

## 2. Materials and Methods

### 2.1. Animals

The experimental procedures were performed under the oversight and approval of the UKM Animal Ethics Committee, with the approval number FSK/2022/KHAIRUL OSMAN/20-JULY/1262-AUG.-2022-AUG.-2024. Twenty-four male Sprague Dawley rats weighing 200 to 250 g, aged 6 to 8 weeks, were obtained from the Animal House at Universiti Kebangsaan Malaysia (UKM). The rats were kept in a controlled setting in the animal facility, with the temperature maintained at 22 ± 3 °C. They were subjected to a lighting schedule of 12 h of light followed by 12 h of darkness. The subjects were supplied with palletized meals purchased from the UKM Animal House and water ad libitum.

The rats were randomly divided into one control group and three test groups, each housed individually in plastic cages measuring 29 cm × 43 cm × 16 cm. To mimic real-world Wi-Fi usage—where exposure is not in close proximity to the device—and to eliminate thermal effects, the cages were placed 20 cm away from a TP-LINK AC750 Wireless Dual Band Wi-Fi Router (Archer C20, Shenzhen, China) [16]. The router emitted RF-EMR at a frequency of 2.45 GHz, compliant with the IEEE 802.11n standard. An Electromagnetic Radiation Tester was used to ensure the frequency remained stable throughout the experiment. The experimental room had aluminum foil covering its walls to provide a controlled RF-EM Wi-Fi environment. This served as a barrier against external RF-EM Wi-Fi transmissions.

This study involved subjecting the rats to uninterrupted exposure to RF-EM Wi-Fi radiation for eight weeks. At the end of these eight weeks, all the rats were executed ethically by administering a lethal combination of Ketamine, Xylazine, and Zoletil through an intraperitoneal injection. The confirmation of the rats’ death was determined using different indications, such as the absence of the righting reflex, expulsion of cardiac function, poor response to tail pinching, loss of withdrawal response in both forelimbs and hindlimbs, and no corneal reflex. Later, sperm and testis samples were obtained from each rat.

### 2.2. Study Groups

Following a one-week acclimation period, the animals were randomly allocated into four equal groups, each containing six rats (*n* = 6). Over an 8-week period, each group was exposed to a 2.45 GHz Wi-Fi device under different conditions [17]:

- Control: non-operating device;
- Test 1: device operating for 4 h daily;
- Test 2: device operating for 8 h daily;
- Test 3: device operating for 24 h daily (continuously).

### 2.3. Testis and Epididymis Collection

The testes and epididymis were carefully dissected. Gauze and pre-warmed PBS solution at 37 °C were employed to delicately eliminate any surplus blood from the area. Before accurate measurement, the adjacent adipose tissue was also removed. Subsequently, the epididymis and testes were both accurately weighed. Afterwards, the testes were submerged in a 10% neutral formalin fixative solution for subsequent handling.

### 2.4. Reproductive Organ Coefficient

The organ coefficient of each dissected reproductive organ was calculated using the following formula: the wet weight of the organ (g)/body weight (g) × 100

### 2.5. Serum Collection

Blood was taken through cardiac puncture after the rats were euthanized. The samples were placed in blood tubes and left for 30 min at 37 °C. Subsequently, the samples were centrifuged at 1500× g for 10 min at 4 °C. The serum was pipetted, placed in Eppendorf tubes, and labelled. The serum was then frozen at −80 °C until the analysis was conducted.

### 2.6. Sperm Collection

Sperm specimens were collected from the cauda epididymis minced in pre-warmed PBS (ThermoFisher Scientific, Waltham, MA, USA). These samples were then subjected to a 30 min liquefaction period at a stable temperature of 37 °C, facilitating sperm swim-up from the epididymal tubules.

### 2.7. MDA Analysis

An MDA ELISA kit (Elabscience, Wuhan, China) was used with a previously prepared serum. All protocols followed the manufacturer’s recommendations. The ELISA plate was then read using a SpectraMax Plus 384 Microplate spectrophotometer (Molecular Devices, San Jose, CA, USA) at a wavelength of 450 nm.

### 2.8. Sperm Parameters

#### 2.8.1. Sperm Concentration

A 10 µL sperm suspension was placed onto a Makler Chamber (Sefi Medical Instruments Ltd., Haifa, Israel). The sperm concentration was calculated using the average of the counts obtained from ten grid cells examined under a bright field microscope (Olympus CH-2, Tokyo, Japan) with a 20× magnification. Following standard counting rules to avoid double counting, only sperm heads entirely within each square or touching the right and bottom borders were included. These 10 squares represented the sperm count in 10⁶/mL.

#### 2.8.2. Sperm Motility

Sperm motility was evaluated according to the protocols the World Health Organization (WHO) established in 2010. The categorization of sperm motility was based on groups: Group A as progressive motility, Group B as non-progressive motility, and Group C as immotile. To carry out the assessment, a 10 µL volume of sperm suspension was placed onto a microscope slide and then covered with a slip. Two hundred cells were observed and recorded in duplicate using a bright-field microscope (Olympus CH-2, Tokyo, Japan) with 40× magnification. The percentage of sperm motility was determined as (A + B)/total counted sperm × 100.

#### 2.8.3. Sperm Viability

An assessment of sperm viability was conducted through the hypo-osmotic swelling test (HOST). The hypo-osmotic swelling solution was prepared by dissolving 0.735 g of sodium citrate dehydrate (Sigma Aldrich, Steinheim, Germany) and 1.351 g of D-fructose (Sigma Aldrich, Steinheim, Germany) in 100 mL of distilled water. A sperm suspension was mixed with the hypo-osmotic swelling solution in a 1:10 ratio. This mixture was then incubated at 37 °C for 30 min. Subsequently, about 10 µL of the mix was smeared on a microscope slide and allowed to air dry at room temperature. To enhance the visibility of the sperm for bright-field microscope observation, the smear was stained using the Diff Quick staining method. After staining, the slides were rinsed and allowed to air dry. Viable sperm were then counted under 40× magnification, with 200 sperm cells counted in duplicate.

### 2.9. Histopathology of Testes

#### 2.9.1. Tissue Processing and Sectioning

The right testis of each rat was processed using the Excelsior AS tissue processor (Thermo Scientific, Waltham, MA, USA) and then transferred to paraffin for block formation using a tissue embedding machine (Thermo Scientific Histostar, Waltham, MA, USA). The immersion was as follows: 70% Ethanol, 95% Ethanol, 100% Ethanol (3×), Ethanol/Toluene, Toluene (3×), and paraffin (3×). Tissue blocks were sectioned using a microtome (Leica Microsystems, Wetzlar, Germany) at a thickness of 3 µm. These sections were placed on a water bath at 40 °C and then mounted on glass slides for H&E (Hematoxylin and Eosin) staining.

#### 2.9.2. H&E Histological Staining

- Deparaffinization Process: Previously prepared glass slides were air-dried using an XH-2001 tissue dryer (C&A Scientific-Premiere, Sterling, VA, USA) at 60 °C until completely dry. Subsequently, the deparaffinization process was carried out via immersion in Xylene (2×), 100% Ethanol, 80% Ethanol, and 70% Ethanol.
- H&E Staining Process: The H&E staining process was performed accordingly: Hematoxylin, tap water, Xylene, tap water, Scott’s tap water, tap water, and Eosin.
- Dehydration and Clearing Process: The Dehydration and Clearing process was carried out as mentioned: 80% Alcohol, 90% Alcohol, 100% Alcohol (2×), and Xylene (2×).

Subsequently, the slides were air-dried before mounting. ImageJ image analysis software v1.45 (Madison, WI, USA) was employed to quantify the Johnsen Score [18], epithelial diameter, and height within the seminiferous tubules per individual rat. Twenty seminiferous tubules from each testis were subjected to analysis (duplicated).

### 2.10. Statistical Analysis

A one-way analysis of variance (ANOVA) with Tukey’s post hoc analysis was performed using IBM SPSS Statistics 27 (Chicago, IL, USA) to evaluate the differences between the control group and the three test groups (test 1, test 2, and test 3). This analysis was conducted after confirming that the Kolmogorov–Smirnov test for normality yielded *p* > 0.05, indicating adherence to a normal data distribution. This study reports all values in the format of mean ± SEM (Standard Error of the Mean). When the data deviated from normality assumptions (Kolmogorov–Smirnov: *p* < 0.05), the Kruskal–Wallis H test was employed as an alternative method. Statistical significance was determined based on the ANOVA or Kruskal–Wallis H test results, with significance established at *p* < 0.05.

## 3. Results

### 3.1. MDA Level

**Figure 1:**
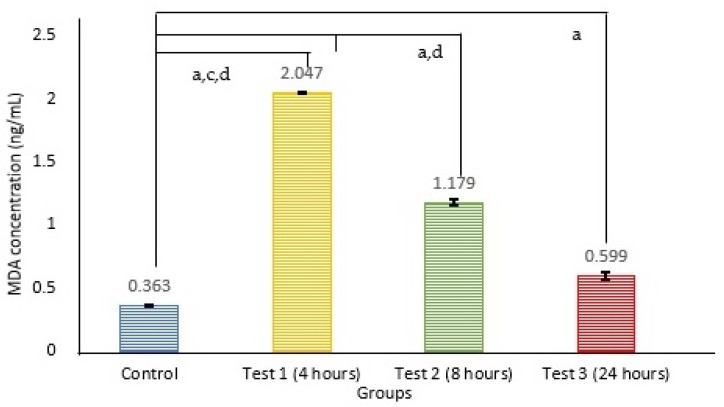
The data are expressed as the mean ± SEM for n = 6, representing the MDA levels measured for each group. a indicates a significant difference compared to the control group, c indicates a significant difference with test 2, and d indicates a significant difference with test 3.

An analysis was undertaken to distinguish the variance in MDA levels (depicted in Figure 1) among the four distinct groups. The results of a one-way ANOVA revealed a significant distinction among the groups concerning MDA levels after exposure to 2.45 GHz radiation with varying exposure durations (F (3, 3.373) = 1513.204, *p* < 0.001). A post hoc Tukey analysis demonstrated significant differences between test group 1, test group 2, test group 3, and the control group (F (3, 20) = 1513.204, *p* < 0.001). The MDA level peaked for 4 h of exposure, followed by 8 h and 24 h. Interestingly, as the exposure duration increased, the MDA level gradually decreased, approaching that of the control group.

### 3.2. Reproductive Organ Coefficient

A one-way analysis of variance (ANOVA) was conducted to examine the variations in reproductive organ coefficients among the four unique groups: control, test 1, test 2, and test 3. Referring to Table 1, the results revealed significant differences among the three test groups regarding the right testis coefficient (F(3, 20) = 8.47, *p* < 0.01). A subsequent post hoc Tukey analysis demonstrated that only test 1 and test 2 exhibited statistically significant differences when compared to the control (F(3, 20) = 8.47, *p* < 0.01). Likewise, a considerable difference emerged among the three test groups regarding the left testis compared to the control (F(3, 20) = 6.10, *p* < 0.05). A subsequent post hoc Tukey analysis revealed that test 1 and test 2 displayed statistically significant differences when compared to the control (F(3, 20) = 6.10, *p* < 0.05).

### 3.3. Sperm Parameters

**Figure 2:**
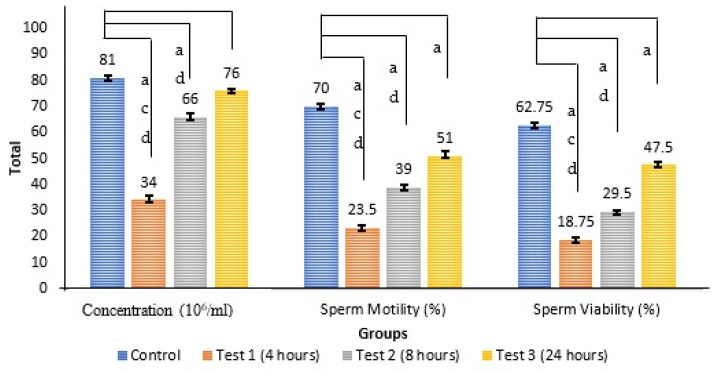
The data are shown as the mean ± SEM of *n* = 6 for the sperm concentration (mL), sperm motility (%), and sperm viability (%) in each group. a indicates a significant difference compared to the control group, c indicates a significant difference with test 2, and d indicates a significant difference with test 3.

The data are presented in Figure 2.

#### 3.3.1. Sperm Concentration

This study revealed a notable difference in sperm counts among the three groups compared to the control (F(3, 20) = 103.422, *p* < 0.001). Through a post hoc Tukey analysis, it was found that test 1 and test 2 significantly differed from the control (F(3, 20) = 103.422, *p* < 0.001). Therefore, it may be inferred that the duration of exposure of 4 h resulted in a decline in sperm counts. However, sperm counts were restored at 8 h and had nearly returned to their baseline values at 24 h.

#### 3.3.2. Sperm Motility

The analysis yielded significant differences among the three test groups compared to the control (F(3, 20) = 209.388, *p* < 0.001). A post hoc Tukey analysis confirmed a significant difference between the control and test 1, test 2, and test 3 (F(3,20) = 103.422, *p* < 0.001). It was concluded that exposure for 4 h, 8 h, and 24 h decreased the percentage of motile sperm. Interestingly, the rate of motile sperm increased after additional exposure; specifically, an improvement began at 8 h, with further enhancement observed at 24 h.

#### 3.3.3. Sperm Viability

There was a significant difference among the three test groups compared to the control (F(3, 20) = 254.276, *p* < 0.001). A post hoc Tukey analysis was performed and found that test 1, test 2, and test 3 were significantly different from the control (F(3, 20) = 254.276, *p* < 0.001). In conclusion, 4 h of exposure resulted in the lowest viability, followed by 8 and 24 h. Hence, it is evident that throughout the 24 h exposure, there was a notable increase in the percentage of viable sperm; however, this increase began at 8 h.

### 3.4. Correlation Between MDA and Sperm Parameters

**Figure 3:**
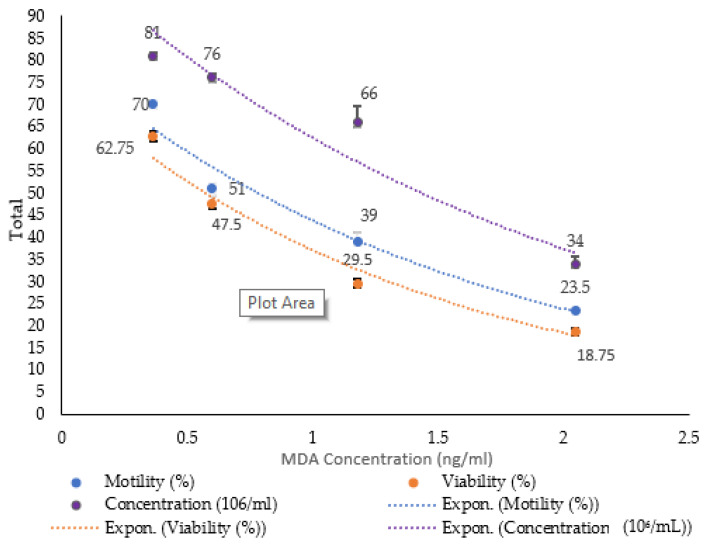
This graph shows the correlations between the MDA level and the sperm concentration (X10^6^), motility (%), and viability (%). The data are reported as the mean ± SEM, with n = 6 for each group.

The data are presented in Figure 3.

Pearson correlation coefficients were obtained to evaluate the relationships between MDA and sperm concentration (X10^6^), motility (%), and viability (%). The MDA level was more strongly negatively related to the viability (%) (r (22) = −9.69, *p* < 0.01) than to the sperm concentration (X10^6^) or motility (%) (r (22) = −9.55, *p* < 0.01). Therefore, we can conclude that all the variables showed strong negative correlations with MDA levels. As MDA levels rose, there was a tendency for sperm parameters to decline. Notably, MDA levels peaked at the 4th hour before decreasing at the 8th and 24th hours. Correspondingly, sperm parameters decreased initially at the 4th hour, followed by further declines at 8 and 24 h. By 24 h, the MDA levels and sperm parameters were near those of the control group.

### 3.5. Histopathology of Testes

**Figure 4:**
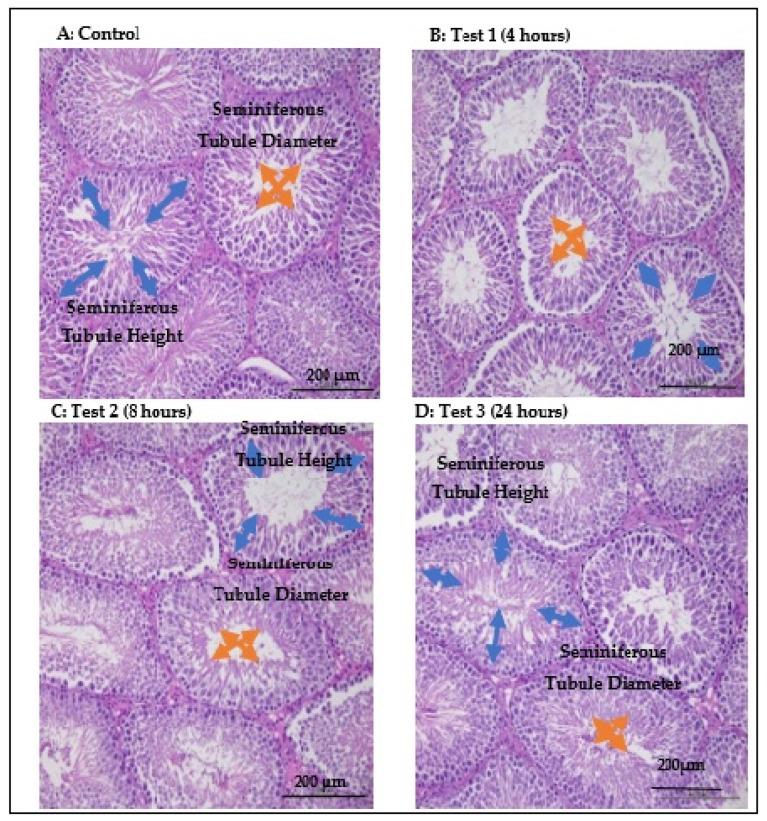
Histopathology of testes for each group (200 µm) using H&E staining: (**A**): control; (**B**): test 1 (4 h); (**C**): test 2 (8 h); and (**D**): test 3 (24 h). The orange arrow showed the seminiferous diameter and the blue arrow showed the seminiferous tubule height.

**Figure 5:**
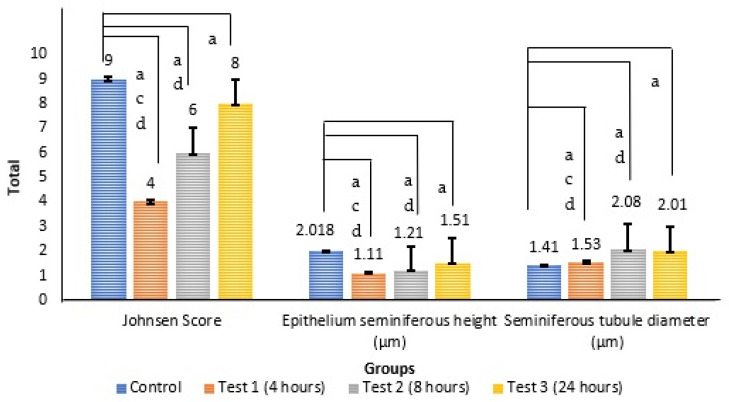
The data are presented as the mean ± SEM for the Johnsen Score, seminiferous epithelium height, and seminiferous tubule diameter, with a sample size of *n* = 6 for each group. a indicates a significant difference compared to the control group, c indicates a significant difference compared to test group 2, and d indicates a significant difference compared to test group 3.

The analysis was based on Figure 4 and was performed using Image J. The data are presented in Figure 5.

#### 3.5.1. Johnsen Score

The Kruskal–Wallis H test unveiled a statistically significant discrepancy in Johnsen scores among the three groups, with an H value of 395.757 and *p* < 0.001. The control group boasted the highest mean rank at 378.55, followed by test group 3 at 339.64, test group 2 at 166.82, and test group 1 with the lowest mean rank of 4.39. This suggests a decrease in Johnsen scores following exposures of 4 h, 8 h, or 24 h. Yet, with prolonged exposure, the Johnsen scores gradually improved at 8 h and approached normal levels at 24 h.

#### 3.5.2. Seminiferous Tubule Diameter

The Kruskal–Wallis H test revealed a significant disparity among the three groups regarding the seminiferous tubule diameter (H (3) = 128.023, *p* < 0.001). Test group 2 had the highest mean rank at 307.35, followed by test group 3 at 304.86, test group 1 at 202.74, and the control group with the lowest rank at 138.49. Consequently, it can be inferred that the most substantial increase in the seminiferous tubule diameter occurred at 8 h, followed by 24 h and 4 h.

#### 3.5.3. Seminiferous Epithelium Height

A one-way ANOVA showed a notable distinction among the three groups (F (3, 464) = 304.966, *p* < 0.001). A post hoc Turkey analysis revealed significant differences in test group 1 (2.45 GHz/4 h), test group 2 (2.45 GHz/8 h), and test group 3 (2.45 GHz/24 h) compared to the control group (0 MHz/24 h) (F (3, 20) = 304.966, *p* < 0.001). The minimum height of the seminiferous epithelium was noted after the 4 h exposure. This was followed by 8 and 24 h. With the 24 h exposure duration, the seminiferous epithelium height in the test groups gradually increased, converging towards the level observed in the control group.

## 4. Discussions

The current study evaluated the effects of 2.45 GHz Wi-Fi exposure on sperm parameters, specifically the count, viability, and motility, at various intervals. Comparisons with the previous literature on EMF exposure and male fertility highlight the significance of considering exposure time as a critical variable. While several studies have found detrimental effects on sperm qualities, others have found no significant effect. This outcome highlights the complexities of the relationship between RF-EMR exposure and male reproductive well-being.

Our research aimed to unfold this mystery by investigating the impact of Wi-Fi presence in a time-dependent environment. Our study showed that the effects of the RF-EMR exposure time on sperm parameters and testicular histopathology were significantly different from one group to another. Surprisingly, exposures lasting four hours resulted in more apparent deleterious effects than exposures lasting eight or twenty-four hours. Specifically, we found that after the four-hour exposure period, there was a significant decrease in the number of sperm and their viability and motility. Additionally, there was an increase in histological abnormalities in the testicular tissue. According to these findings, the prevalent assumption that longer exposure durations correlate with more deleterious effects is called into question. These data also show that there may be a threshold beyond which RF-EMR exposure becomes more damaging. As the damage becomes severe, body defenses try to recover from the injuries.

### 4.1. MDA

Exposure to RF-EMR prompts an investigation into MDA levels, which serve as a biomarker of oxidative stress in testicular tissue. A consistent pattern emerges across various studies, indicating a notable elevation in MDA levels after exposure.

For instance, Shahin et al. observed heightened MDA levels following daily exposure lasting 3 h over 30 days [19]. Similarly, Qin et al. documented increased MDA concentrations after exposures ranging from 1 to 3 h daily for 28 days, with pronounced increments observed particularly at the 2 and 3 h intervals [11]. Jonwal et al. reported parallel findings, with MDA levels exhibiting an escalation after 2 h of daily exposure for 30 days [20]. These observations were echoed by El-Bediwi et al., who noted elevated MDA levels following 2 h of exposure conducted over a 28-day timeframe [21]. Furthermore, studies conducted by Saygin et al., and Yahyazadeh et al. further support this trend, revealing heightened MDA levels after exposures lasting 1, 2, 3, and 4 h [12,22].

Meanwhile, our research contributes to this body of knowledge by observing MDA levels peaking at 4 h post-exposure and subsequently exhibiting a decline at the 8 and 24 h marks, indicative of a potential adaptive mechanism aimed at attenuating lipid peroxidation. The MDA level correlates positively with a reduction in sperm parameters. Higher levels of MDA result in a more substantial disturbance of sperm parameters. At the 4 h mark, the MDA level reached its highest point, corresponding with the most significant drop in sperm parameters. The MDA level subsequently decreased at 8 and 24 h, mainly corresponding to improvements in sperm parameters.

### 4.2. Sperm Parameters

Multiple studies have explored the impact of RF-EMR on sperm characteristics, utilizing rodent models to assess parameters such as the count, motility, and viability [23,24,25,26]. The precise processes underlying these impacts are not yet fully known, but the findings repeatedly point to the possibility of harmful effects on sperm quality. To bridge this knowledge gap, researchers have used a range of exposure durations, from 30 min to 24 h, to cover periods where sperm parameters may deteriorate the most [15,27,28].

The results of Shokri et al.’s study, where participants were exposed to 2 h of daily radiation over 30 days, showed reductions in sperm count and viability [24]. Mohamed et al. also observed a decrease in sperm count following exposure of 2 h a day for 50 days [29]. This pattern aligns with subsequent findings from Qin et al., who found that exposure for 28 days, with daily durations ranging from 1 to 3 h, led to decreased sperm counts at 2 and 3 h intervals, with no significant changes observed in motility or viability [11]. Furthermore, Shahin et al.’s investigation revealed diminished sperm counts, motility, and viability over 28 days with 2 h of daily radiation exposure [23]. Interestingly, Farag et al.’s study, which involved 1 h of daily radiation exposure over 56 days, showed a significant decline in sperm counts [27]. The same trend persisted in a study by Gevrek et al., where exposure to RF-EMR for 4 h daily over 45 days also decreased sperm counts [25].

In contrast, a study by Owjfard et al. utilized 2 h daily of RF-EMR over 90 days, noting a significant increase in sperm motility [30]. Additionally, Dong et al. made intriguing observations regarding fluctuations in sperm parameters after exposure to RF-EMR for just 30 min [28]. Six hours post-exposure, there was an increase in the number of actively moving sperm cells, along with higher curvilinear velocity, linear velocity, and average route velocity. The absence of a definitive conclusion may be attributed to the limitation of utilizing only a singular exposure time. Hence, comparing various exposure durations would provide a more comprehensive understanding.

Our study sheds light on this aspect, revealing that a 4 h exposure duration elicited the most adverse effects on sperm parameters, with a gradual improvement observed at 8 h and values nearing those of the control by 24 h. This finding aligns with the results documented by Yahyazadeh et al., who similarly noted a decline in sperm counts at 2, 4, and 6 h, followed by a subsequent increase at 8 h [22]. This underscores the potential existence of a reparative mechanism following a particular duration of exposure. Exposure to RF-EMR can lead to oxidative stress, affecting male reproductive health by disrupting the balance between ROS production and antioxidant defenses [11,12]. Notably, an 8 h exposure period may bolster antioxidants, countering ROS production. Moreover, this phenomenon is linked to the activation of DNA repair mechanisms in response to oxidative stress, which helps mitigate the adverse effects on male germ cells during recovery [31].

### 4.3. Histopathology of Testes

Regarding testicular histopathology, conflicting results from various studies using varying radiation exposure times have been observed, creating a patchwork of ambiguous results. While some exposure times have been associated with negative consequences, such as reductions in the diameter and height of the seminiferous tubule epithelium, inconsistent results have been reported for other exposure times, with some studies indicating no discernible effect [23,26,28,32].

Syntheses of findings from the reviewed studies consistently underscores a concerning trend regarding the impact of RF-EMR exposure on testicular tissue integrity and functionality. These findings began with the observations of Pandey et al., who noted significant declines in the seminiferous tubule diameter and germinal epithelium height following controlled EMF sessions [33]. Subsequent investigations by El-Bediwi et al. further elucidated this cascade of detrimental effects [21]. Their findings revealed compromised spermatogenesis, marked by reduced spermatogenic cell counts and instances of necrosis within seminiferous tubules, across individuals exposed to EMF for varying durations. Delving deeper into the histopathological repercussions of sustained EMF exposure, Shokri et al. provided insights into a spectrum of degenerative changes affecting seminiferous tubules and Leydig cells [24]. Qin et al. corroborated this progressive tissue deterioration and highlighted multiple tissue degeneration characterized by cytoarchitectural anomalies and cellular necrosis [11]. The severity of each effect escalated with prolonged exposure durations.

Subsequent studies by Shahin et al., Shahin et al., and Yahyazadeh et al. consistently echoed these findings, emphasizing reductions in spermatogenesis and observable manifestations such as germinal epithelial sloughing, vacuolation, and notable declines in both the tubular diameter and epithelial thickness, all indicative of compromised testicular health attributed to EMF exposure [19,22,23]. In a poignant observation, Yahyazadeh et al. underscored the severity of these effects by highlighting the observed pronounced histopathological alterations, including vacuolization, necrosis, and disruptions in interstitial tissue integrity [22]. This observation further emphasizes the profound impact of EMF on testicular histology. However, amidst these trends, Dong et al. presented a nuanced perspective, reporting no significant changes in testicular tissue morphology in their study cohort following RF-EMR exposure [28].

As previously mentioned, the inconclusive outcomes of research studies can often be attributed to the duration of exposure. Our research contributes to this discourse by revealing that the testicular histopathology was most adverse after 4 h of exposure, improved by 8 h, and nearly reached control levels by 24 h. As mentioned earlier, the 8 h exposure period appears to have significantly impacted antioxidant levels, potentially suppressing further production of ROS. Our findings indicated that levels of MDA, a marker of lipid peroxidation, peaked at 4 h but then decreased notably at 8 and 24 h. This suggests a possible initiation of lipid peroxidation recovery by antioxidants around the 8 h mark.

Furthermore, reproductive hormones such as luteinizing hormone (LH) and testosterone could have played a role. Studies by Yahyazadeh et al., Gevrek et al., and Stephen et al. showed decreases in LH and testosterone levels following 4 h of exposure [22,25,34]. We hypothesize that by the 8 h mark, a negative feedback mechanism was triggered, resulting in an increase in LH and testosterone, initiating recovery. This suggests a potential interplay between antioxidant activity, lipid peroxidation, and reproductive hormone regulation during the 8 h exposure period.

### 4.4. Organ Coefficient

Like those investigating sperm parameters and testicular histology, studies investigating organ coefficients following RF-EMR exposure have shown consistent trends in some cases but varying outcomes in others. The relationship between RF-EMR exposure and the testis organ coefficient remains complex and not yet fully understood. Our study observed that testis weights were lowest at 4 and 8 h exposure durations but increased at 24 h. This suggests that RF-EMR had a more pronounced negative effect at 4 and 8 h, while the 24 h exposure group showed signs of recovery. These findings align with our MDA results, where MDA levels were higher at 4 and 8 h compared to 24 h, indicating higher oxidative stress with shorter exposure durations. Supporting our findings, Dasdag et al. also reported a reduction in testis weight after exposure to 2.45 GHz RF-EMR for 24 h [35]. The variability in results across different studies may be attributed to differences in the experimental design, exposure parameters, and biological responses. Further research is necessary to clarify the conditions under which RF-EMR exposure affects the testis organ coefficient and to elucidate the underlying mechanisms.

## 5. Conclusions

There are inconclusive findings regarding the effects of RF-EMR on the male reproductive system, and it is plausible that variations in exposure times and the use of a single duration may contribute to this ambiguity. To address this, our study employed three distinct exposure durations to elucidate potential differences. Our findings revealed that male rats exposed to continuous Wi-Fi radiation for 4 h exhibited diminished fertility due to cellular damage. Following an 8 h exposure, the rats’ defense mechanisms were activated, thus mitigating further injury. However, complete recovery remained elusive. This study was limited to investigating the effects of a single frequency (2.45 GHz) and did not consider other Wi-Fi frequencies or combined exposures. Future studies should explore different frequencies, combined exposures, and intensities, as well as the molecular mechanisms of cellular damage and recovery, to provide a more comprehensive understanding of the impact of RF-EMR on male reproductive health.

## Figures and Tables

**Table 1:** Organ coefficient (testis).

Group/Organ	Control	Test 1 (4 h)	Test 2 (8 h)	Test 3 (24 h)
Testis (right)	0.32 ± 0.01	0.18 ± 0.02 ^a^	0.18 ± 0.03 ^a^	0.32 ± 0.04
Testis (left)	0.32 ± 0.01	0.19 ± 0.02 ^a^	0.18 ± 0.03 ^a^	0.30 ± 0.04

The organ coefficient (testis) data are presented as the mean ± standard error of the mean (SEM), with a sample size of 6 per group. ^a^ A significant difference was noted in the testis relative to the control group.

## Data Availability

Data are contained within the article.
